# Larvicidal effects of a neem (*Azadirachta indica*) oil formulation on the malaria vector *Anopheles gambiae*

**DOI:** 10.1186/1475-2875-6-63

**Published:** 2007-05-22

**Authors:** Fredros O Okumu, Bart GJ Knols, Ulrike Fillinger

**Affiliations:** 1 University of Nairobi, School of Biological Sciences, P.O. Box 30197 00100 GPO Nairobi Kenya; 2 Ifakara Health Research and Development Centre, Public Health Entomology Unit, P.O. Box 53 Kilombero Tanzania; 3 Laboratory of Entomology, Wageningen University and Research Centre, P.O. Box 8031, 6700 EH, Wageningen, The Netherlands; 4 Durham University, School of Biological and Biomedical Sciences, South Road, Durham, DH1 3LE, UK

## Abstract

**Background:**

Larviciding is a key strategy used in many vector control programmes around the world. Costs could be reduced if larvicides could be manufactured locally. The potential of natural products as larvicides against the main African malaria vector, *Anopheles gambiae s.s *was evaluated.

**Methods:**

To assess the larvicidal efficacy of a neem (*Azadirachta indica*) oil formulation (azadirachtin content of 0.03% w/v) on *An. gambiae s.s*., larvae were exposed as third and fourth instars to a normal diet supplemented with the neem oil formulations in different concentrations. A control group of larvae was exposed to a corn oil formulation in similar concentrations.

**Results:**

Neem oil had an LC_50 _value of 11 ppm after 8 days, which was nearly five times more toxic than the corn oil formulation. Adult emergence was inhibited by 50% at a concentration of 6 ppm. Significant reductions on growth indices and pupation, besides prolonged larval periods, were observed at neem oil concentrations above 8 ppm. The corn oil formulation, in contrast, produced no growth disruption within the tested range of concentrations.

**Conclusion:**

Neem oil has good larvicidal properties for *An. gambiae s.s*. and suppresses successful adult emergence at very low concentrations. Considering the wide distribution and availability of this tree and its products along the East African coast, this may prove a readily available and cheap alternative to conventional larvicides.

## Background

Malaria in sub-Saharan Africa can be controlled by attacking its prime vectors, notably *Anopheles gambiae s.l*. Since the onset of mosquito control activities in the early 1900s, several challenges continue to hinder efforts to effectively control malaria. These include insecticide resistance, limited access to essential resources (human, capital, and equipment) that affect conventional use of control methods, and insect adaptation and altered behavioural traits, such as exophily and exophagy [1]. The need to develop and incorporate new alternative tools for integrated vector management remains key, where methods to reduce adult biting and control of aquatic stages are used in combination. Use of larvicides, which dates back to as early as 1899, when Ronald Ross applied kerosene on anopheline larval breeding sites in Sierra Leone [2], is an approach with great potential for future malaria vector control [3]. It is worth emphasizing that although larval control is not widely used in the tropics today, in the past the greatest achievements in malaria control were based on the use of larvicides, for example the eradication of *An. gambiae *from Brasil [4] and *Anopheles arabiensis *from Egypt [5].

At present, mosquito larvicides include organophosphates, insect growth regulators and microbial larvicides. Current research focuses on microbials such as *Bacillus thuringiensis *var. *israelensis (Bti) *and *Bacillus sphaericus *[6,7] as well as on botanicals with larvicidal, oviposition inhibiting, repellent or insect growth regulatory effects [8,9]. Such products contain a multitude of active ingredients with different modes of action, which lessens the chance of resistance developing in mosquito populations. The neem plant (*Azadirachta indica*) and its derived products have shown a variety of insecticidal properties on a broad range of insect species [10,11].

Neem products have been shown to exhibit a wide range of effects that are potentially useful for malaria control and include antifeedancy [9], ovicidal activity, fecundity suppression [12], insect growth regulation [13,14] and repellency [15-17]. These effects are frequently attributed to the azadirachtin contents of the products [10,13]. Recent studies have also demonstrated neem-induced effects on vitellogenesis and severe degeneration of follicle cells during oogenesis in mosquitoes [9]. It has been argued that the pesticidal efficacy, environmental safety, and public acceptability of neem and its products for control of crop pests would ensure its adoption into mosquito control programmes [12,18]. Presently, however, none of the commercially available neem formulations, which include emulsifiable concentrates (ECs), wettable products (WPs), suspension concentrates, ultra low volume (ULV) and granular formulations, are used for this purpose.

Neem-based products are relatively safe towards non-target biota, with only minimal risk of direct adverse effects on aquatic macro invertebrates resulting from contamination of water bodies with neem-based insecticides [19-21]. In addition, the products are less likely to induce resistance due to their multiple modes of action on insects [22]. Research on neem products for the control of arthropods of medical and veterinary importance has been ongoing for some time. Various studies have focused on the culicine species *Culex tarsalis *and *Culex quinquefaciatus *[12,18,22,23], besides *Aedes aegypti *[24-26]. There have also been studies that assessed the larvicidal potential of neem products on anophelines, notably *Anopheles culicifacies*, *An. arabiensis*, *An. gambiae *and *Anopheles stephensi*. [9,22,27,28]. The current studies aimed to determine the larvicidal potency of an emulsified neem oil formulation (32% neem oil) against *An. gambiae s.s*., which is one of the most notorious malaria vectors in sub-Saharan Africa.

## Materials and methods

### Mosquitoes

The *An. gambiae s.s*. larvae used in this study were from a colony established in 2001 at the Thomas Odhiambo campus of the International Centre of Insect Physiology and Ecology (ICIPE), Mbita Point, western Kenya. Mosquitoes were reared under semi-natural conditions in a greenhouse, following standard operating procedures for mosquito maintenance [29-31].

### Oil formulations

Two experimental formulations, both of which were emulsified concentrates, were tested and compared. The test formulation was an emulsified concentrate, containing 32% neem seed oil (an equivalent of 0.03% azadirachtin), an emulsifier (5%) and 63% isopropanol (solvent). The neem oil was extracted from seeds collected in coastal Kenya. A corn oil formulation with similar solvent and emulsifier contents and proportions was used as the control formulation.

### Experimental procedures

The larvicidal effects of the neem oil formulation were tested on *An. gambiae s.s*. under greenhouse conditions [32]. Baseline tests were initially run in distilled water to determine the range of lethal doses of the formulation [33]. The maximum dosage of the neem formulation to be applied was determined as 32 ppm, as this resulted in high larval mortality within days. In the main experiment, the larvae were reared in 15 × 20 cm plastic trays. Stock solutions of 1,000 ppm (0.1%) of the two experimental formulations were prepared. Six aliquots were prepared from this stock solution to obtain concentrations of 0.5, 2, 4, 8, 16 and 32 ppm respectively. A fresh stock solution was prepared for each replicate experiment. X ppm in this case refers to X parts of the experimental formulation mixed with (1.000.000-X) parts of the ordinary larval breeding medium. Thus each of the six trays had the ordinary larval rearing medium supplemented with the neem formulation at the different concentrations (i.e. 0.5 to 32 ppm). The same method of application was used for the corn oil formulation. Fifty 3^rd ^to 4^th ^instar larvae were introduced carefully into each tray which were then topped up to 1 L. A negative control was run in freshly collected water from Lake Victoria, routinely used to rear larvae of the colony.

The larvae in all the trays were fed every 24 hrs on equal amounts of Tetramin^® ^Baby fish food using a 'dip stick' (approx. 0.015 g). Tetramin^® ^Baby is a powdered diet and spreads evenly across the water surface. Six replicates were run under the same microclimatic conditions. The mortality of the larvae was monitored every 24 hrs. All the pupae were collected, counted and kept in labeled glass vials capped with cotton wool. The solution with which the pupae were collected was also kept in the same vials such that the pupae remained under the same experimental conditions and concentrations as during the larval stages. The pupae were further monitored for 24 to 48 hours when emerging adults were counted and recorded. Larvae were observed during their entire lifespan, in order to monitor the usually delayed effects of neem products [34,35].

Percentage cumulative pupation and the mean larval periods were calculated for all concentrations of both the corn oil and neem oil formulation. The mean larval periods for each tray were determined using the following formula:

((A*1) + (B*2) +(C*3) + (D*4) .........+ (H*8))/total number of pupae collected

where A, B, C, D......H are the number of pupae that were collected on days 1,2,3,4 to 8 respectively. The logical argument in this formula is that the larvae which pupated after a particular number of days had actually lived that same number of days. The larval period is summed across the third and fourth larval instars. Growth indices of the larvae were determined as the ratio between percent pupation and the mean larval periods [23].

Percentage emergence inhibition was determined as 100-A where A was the % successful emergence. Emerging adults were grouped age-wise whenever they emerged, and kept in different cages. Adults emerging from each experimental tray were kept separately. This set up was used to study the sublethal effects of the treatments that might have been carried over from the aquatic stage treatments. Adult mosquitoes were continuously provided with water and a 6% glucose solution dispensed from clean cotton wool daily. These mosquitoes were kept at room temperature and a photoperiod of 12 hrs light/dark. No further neem oil or corn oil treatments were administered in this phase of the experiment. Adult mortality was recorded every 24 hrs. The mean longevity of these adults was calculated for both sexes and oil formulations at the different concentrations. Longevity was calculated as the total number of days lived by a single adult mosquito from emergence to death. Adults emerging from the negative control trays (no oil) were used as the control group. The maximum number of days lived by the emerged adults from each group was also recorded.

### Statistical analysis

The mortality of the larvae (300 per concentration) in both the neem and corn oil formulation were corrected using Abbott's formula [36], each with the data gained from the negative control. Log-probit analysis [37] was used to determine the median (LC_50_) and 90% lethal concentration (LC_90_). Emergence inhibition (EI) as caused by the two formulations was also corrected with Abbott's formula [36] and the EI_50 _and EI_90 _values determined using probit analyses. The aquatic developmental parameters; growth indices, larval periods and pupation were compared, for both the oil formulations and the negative control, by Analysis of Variance (ANOVA) using Tukey's studentized range test (honestly significant difference test). The longevity of the emergent adults was compared by student t-tests. SAS software was used for the analyses.

## Results

**Figure 1:**
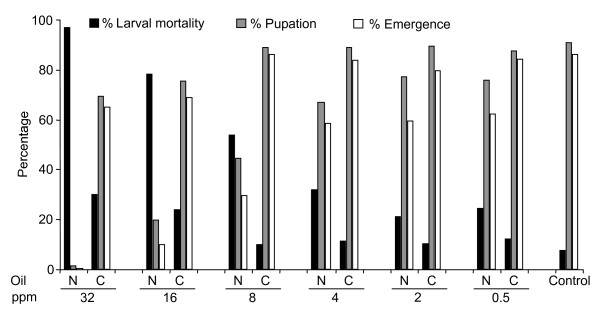
Percentage larval mortality, pupation and adult emergence (as proportion of original numbers tested) of 3^rd^-4^th ^instar larvae of *An. gambiae *following exposure to various concentrations (0.5–32 ppm) of neem oil (N) or corn oil (C). Adult emergence values are percentages of the total number of mosquitoes tested as larvae.

Neem oil was highly larvicidal at high concentrations (32 ppm), but this activity declined progressively as the dose decreased (Figure 1). Corn oil had little if any larvicidal properties at any concentration tested. At concentrations above 16 ppm of the neem oil formulation, over 80% of the observed mortality occurred within the first 72 hrs, while at lower concentrations the rate of mortality was very slow and some larvae, in spite of being 3^rd ^or 4^th ^instar, lived as long as 8 days before they either pupated or died. The median anti-larval potency (LC_50_) of the neem oil formulation after 8 days was 10.7 ppm, and the LC_90 _was 24.1 ppm (Table 1). This was 4.7 times lower than the corn oil formulation, which showed an LC_50 _of 50.7 ppm. The concentration of neem oil that induced median emergence inhibition (EI_50_) was 6.4 ppm while the EI_90 _was 17.4 ppm. Table 1 shows that the EI_50 _value of the neem oil formulation was approximately 8 times lower than that of the corn oil formulation. At 32 ppm the neem formulation inhibited 99.3% of emergence. Whereas the adult emergence steadily increased with decreasing concentrations of the neem oil formulation, there were no observable increments with the corn oil formulation (Figure 1).

**Table 1:** Larval mortality and emergence inhibition of *An. gambiae s.s*. after exposure to neem oil and corn oil formulations.

	Larval mortality †				Emergence inhibition	
	
	192 ‡		72			
	
Oil	LC_50_	LC_90_	LC_50_	LC_90_	EI_50_	EI_90_
Neem	10.7 (7.55–12.15)	24.12 (12.37–37.68)	22.11 (17.14–31.42)	109.34 (65.48–256.20)	6.44 (5.01–7.99)	17.38 (14.62–21.91)
Corn	50.6 (37.96–88.69)	86.85 (62.29–133.67)	58.24 (42.24–121.66)	91.79 (63.54–208.38)	47.28 (14.40–91.27)	79.75 (16.65–135.36)

† All concentrations in parts per million (ppm) with 95% confidence intervals between brackets; ‡ Hours after exposure. Six concentrations were tested and replicated with 50 mosquitoes six times each (n = 300). LC and EI values were determined by probit analysis (Finney 1971). Mortality was corrected using Abbott's formula (Abbott 1925) with values obtained from the negative control.

Table 2 shows the impacts of the two oil formulations on the aquatic growth and development of *An. gambiae s.s*.. Growth indices of the larvae were significantly reduced by neem oil formulation treatments above 8 ppm (P < 0.05). Addition of this formulation to the diet of the larvae reduced their growth indices from 25.8 (in lake water based medium) to as low as 0.5 and 3.6 when applied at concentrations of 32 ppm and 16 ppm; 96% and 86% reductions respectively. The tests also showed that larval development times were significantly prolonged at concentrations equal to or higher than 16 ppm of the neem oil formulation. In addition, pupation was significantly inhibited at concentrations higher than 8 ppm (P < 0.05). When compared with the negative control, the corn oil formulation did not have any significant impacts on either the larval periods, pupation or growth indices of the mosquitoes tested (P < 0.05). Table 3 shows the sub-lethal impacts of the two oil formulations on the emergent adult mosquitoes. When mosquito larvae were exposed to neem treatments in their diet until they pupated and subsequently emerged, sublethal effects on the emergent adults were observed.

**Table 2:** Effects of neem oil and corn oil formulations on pupation, larval growth period and aquatic development rate of *An. gambiae *s.s.

	% Pupation		Larval period (days)		Growth index†	
Oil Concentration (ppm)	Neem	Corn oil	Neem	Corn oil	Neem	Corn oil
32	1.00 c‡	34.83 a	6.42 a	3.25 bc	0.48 d	21.38 bc
16	8.50 c	38.67 a	5.72 ab	3.43 bc	3.58 d	23.28 ab
8	22.50 b	44.67 a	4.62 abc	4.09 abc	10.22 cd	22.33 ab
4	35.00 a	40.50 a	2.87 c	3.58 bc	26.84 ab	23.87 ab
2	37.00 a	43.00 a	4.05 abc	4.03 abc	19.24 bc	21.99 b
0.5	38.17 a	44.00 a	3.73 bc	3.70 bc	21.94 b	23.80 ab
Control	44.50 a		3.50 bc		25.76 ab	

† % pupation/mean larval period. ‡ Numbers in rows/columns without one or more letters in common are significantly different at P < 0.05. Six concentrations were tested and replicated with 50 mosquitoes six times (n = 300) each. In some cases all larvae died (32 ppm of neem oil formulation), and it was assumed that at least one larva lived for at least as long as the experimental period (8 days)

**Table 3:** Effect of sub-lethal concentrations of neem and corn oil formulations on mean (± SD) and maximum (between brackets) adult *An. gambiae s.s*. longevity (in days).

Concentration (ppm)	8		4		2		Control	
	Male	Female	Male	Female	Male	Female	Male	Female
Neem†	17.6 ± 1.8 (26)	19.6 ± 1.7 (27)	19.4 ± 2.1 (26)	20.3 ± 2.1 (26)	20.9 ± 1.9 (26)	21.8 ± 1.6 (31)	22.1 ± 2.7 (38)	27.3 ± 2.4 (42)
Corn	25.1 ± 3.0 (38)	21.6 ± 2.6 (35)	24.6 ± 2.9 (38)	26.2 ± 2.9 (31)	21.2 ± 2.7 (38)	29 ± 1.9 (33)		

† Third and fourth instar larval stages were treated.

The longevity of both male and female adult *An. gambiae s.s*. whose larvae and pupae had been reared in a diet enriched with the neem oil formulation was significantly lower than the longevity of the adults whose larvae and pupae had been reared in the corn oil enriched diet (P < 0.001). At all the tested concentrations, the maximum number of days lived by the emergent adults was significantly higher after the corn oil formulation treatments than after the neem oil formulation treatment (P < 0.001). Generally the adults emerging from neem oil formulation treated trays had a shorter life span than emergent adults from either corn oil formulation treated or the untreated trays. There were no observable sub-lethal effects of the corn oil formulation.

## Discussion

Neem oil was an effective larvicide against *An. gambiae *larvae; it was highly toxic to mosquito larvae and inhibited the development of pupae. The high rates of larval mortality observed at higher concentrations (16 and 32 ppm of the neem oil formulation) within 72 hrs after exposure indicate the high toxicity of the product. The oil is also a potent insect growth regulator (IGR) which led to a 97.5% increase in larval development time and 97.1 % decrease in pupation at 32 ppm when compared to the corn oil and, as a result of the two, there was a 2.2 (8 ppm) to 44.5 (32 ppm) decrease in the growth indices of the insects. These aspects, combined with the emergence inhibition activity ensure that the resultant mosquito population reduction is substantial, even where the larvicidal potential is minimal.

As an emulsifiable concentrate, the neem oil formulation had greatly reduced-sized particles and was evenly mixed within the water column with a few suspended particles on the water surface. The spread of these fine particles probably increased the efficacy of the formulation since *An. gambiae s.s*. are small particle surface feeders. Larval feeding in this species also entails age-dependent and indiscriminate ingestion of any suitably sized particle [38], especially by the larger third and fourth instar larvae. When ingested, the neem product particles induce antifeedancy in larvae either by altering the insect's chemoreception or by reducing the food intake due to its toxicity [13]. Growth disruption was exhibited in both the pupae and the larvae. The percentage emergence in most cases was less than the percentage pupation, which suggests some pupal mortality, although this was not different from the control. The emergence inhibition (EI) values depicted with the neem oil formulation treatments were much lower than the respective lethal concentration (LC) values, an indication that the growth disruption activity of the neem product extended to pupal stages. This additional effect of neem oil ensures that it reduces the overall population of the insects beyond its larvicidal action.

The observation that the action of neem oil formulation was slow and that the neem oil formulation increased the mosquito larval periods was not unusual. Mortality of first instar culicids larvae collected after application of 30 mg/L Margosan-O (an oil based neem seed extract) was 100% after 15 days exposure in pool water [39]. Singh [35] found that a concentration of 32.1 ppm of de-oiled neem seed kernel extract yielded 85% mortality in *Cx. quinquefaciatus *after 12 days of exposure. Considering that our results were obtained by exposing mosquitoes as third and fourth instars, it is likely that treatment of younger instars would lower LC and EI values, thus providing even greater larvicidal potential. A number of studies have also elucidated this trend. For example Boschitz and Grunewald [25] studying *Ae. aegypti *showed age-dependent growth disruption; the sensitivity towards NeemAzal (a neem seed kernel powdered extract with 40% azadirachtin content) decreased with increasing larval age.

The reduction in longevity of the emergent adults indicates that the neem oil formulation had sublethal effects carried over from the larval treatments. This observation is of significance with regard to the afrotropical malaria vector *An. gambiae s.l*., as a reduction in the average adult daily survival rate is key towards lowering its life-time transmission potential [40,41].

Since the control formulation of corn oil had very minimal effects on the mosquitoes, it is certain that the effects described are due to the neem oil and not the emulsifier or solvent. The limited mortality exhibited by the corn oil formulation could have been caused by its oil effects. An important issue with regard to using neem-based products as larvicides is the rapid decay of its active ingredients such as azadirachtin when exposed to sunlight and pH changes [11,13]. Therefore, short term and repeated treatments may be necessary in field applications. This will increase application costs of larval control programmes, but will have the advantage of minimal residual activity and possible side effects.

A comparison of the results presented here with the outcome from various other studies on the efficacy of different neem products is difficult. There are numerous differences with the previous studies, notably because of differences in the origin of products, concentrations of active ingredients of the products, the species of mosquitoes tested, modes of application of the products, and parts of the neem plant from which the products were extracted. Besides this, the actual effective ingredient and its proportionate content are seldom mentioned in most studies. The results obtained in the various studies [9,12,18,25,43,44] are shown in Table 4. These show that various neem products have greatly varying azadirachtin contents, thereby making it impractical to calculate the product potencies with regard to percentages of azadirachtin. Nevertheless, these studies show dramatic impacts of neem formulations on hatch rates, larval development, and emergence inhibition, rendering neem a potentially useful addition to the arsenal of larval control substances.

**Table 4:** Comparison of the effects of various neem-based products and their azadirachtin contents on various mosquito species.

**Product description**	**Azadirachtin content**	**Mosquito species**	**Investigated effects**	**Recorded Potency**	**Larval Sages tested**	**Reference**
Neem oil formulation, An emulsified concentrate made from neem seed oil extracts	0.03% azadirachtin content (32% neem oil)	*An. gambiae*	Larval mortality, IGR and inhibition of adult emergence	LC 50 of 10.68 ppm and EI 50 of 6.44 ppm	3^rd ^and 4^th ^instars larvae and Adults	This article
Neem Azal (Neem seed kernel powdered extract)	34% Azadirachtin A and a total limonoids content of 57.6%	*An. stephensi*	Effects of blood feeding, oviposition, and oocyst ultrastructure	10–1000 ppm treatments impair feeding, oogenesis and oviposition	Adults and oocyst	Lucantoni *et al *2006
Neem Azal (Neem seed kernel powdered extract)	40% Azadirachtin content	*Ae. aegypti*	Larval mortality, molting inhibition	Molting inhibition and larval mortality occurred at all instars	2^nd^, 3^rd ^and 4^th ^instars larvae	Boschitz. And Grunewald 1994
Emulsifiable concentrates		*Cx. tarsalis*	Antifeedancy	5 ppm-10 ppm AZ induces antifeedancy	Adults	Su and Mulla 1998
Neemix EC 4.5	0.0005%-0.001%	*Cx. quinquefasciatus*				
Azad EC 4.5	5 ppm-10 ppm					
Azad WP 10: wettable Product Azad EC 4.5: Emulsifiable concentrate	0.001% 10 ppm	*Cx. tarsalis*	Ovicidal	0% hatching rate observed with Azad WP 10 and 46.7% hatching rate with Azad EC 4.5	Eggs	Su and Mulla 1998
Water based pure neem oil emulsion	Not indicated	*An. stephensi Cx. quinquefasciatus*	Inhibition of Adult emergence	0.1 ml/l of 5% of the neem oil caused 100% emergence inhibition	Imatures (aquatic stages)	Batra *et al *1998
Water based pure neem oil emulsion	Not indicated	*Ae. aegypti*	Inhibition of Adult emergence	0.4 ml/l of 5% of the neem oil caused 100% emergence inhibition	Imatures (aquatic stages)	Batra et al 1998
Pure neem oil made from seed extracts	Not indicated	*Cx. quinquefasciatus Ae. aegypti*	Larval mortality	0.02–0.1% caused 100% larval mortality	4^th ^instar larvae	Sinniah *et al *1994
Neem formulation (name in the original paper is in Arabic)	0.6 ppm-1.9 pm 0.00006% – 0.00019%	*Ochlerotatus japonicus Cx. pipiens pallens*	Larval mortality and inhibition of adult emergence.	LC50 of 0.342 and 0.367 for *Ochlerotatus japonicus *and *Culex pipiens pallens *respectively. 1.9 ppm and 0.6 ppm solutions caused 99% and 75% emergence inhibition respectively	4^th ^Instar Larvae	Mikami and Yamashita 2004

Neem trees are found throughout Africa with a myriad of uses in medicine, pest control, reforestation etc. [42]. The oil can be obtained through pressing (crushing) of the seed kernel both through cold pressing or through a process incorporating temperature controls. The oil yield varies from 25–45% [42]. Its use as a mosquito larvicide will require addition of a surfactant and solvent to ensure equal distribution over water surfaces. Initial experimentation with a neem formulation applied from a knapsack sprayer demonstrated the relative ease with which larval control can take place. Manufacturing of these larvicides can be stimulated through local businesses and does not, unlike current larvicides such as *Bti *or temephos, require importation from outside Africa. With more decentralized and community-based vector control initiatives underway in Africa [45,46] neem-based larvicides may present an ideal option to increase these efforts.

## Conclusion

The neem oil formulation is a highly effective larvicide for anopheline mosquito vector control. Field application for this product may include high pressure knapsack or ultra-low volume (ULV) sprayers ensuring even application. Further studies are necessary to evaluate the optimum dosages for efficient mosquito control under natural field conditions. Non-target effects on other water inhabiting insects, especially mosquito larvae predators also need further investigation.

## Competing interests

The author(s) declare that they have no competing interests.

## Authors' contributions

FO conducted the experiments and drafted the manuscript. UF and BK supervised the experimental work and participated in the writing of the final versions of the manuscript. UF also oversaw the acquisition of laboratory requirements and provision of literature and other logistical issues towards the publication of the article.
